# Distribution Assessments of Coumarins from *Angelicae Pubescentis Radix* in Rat Cerebrospinal Fluid and Brain by Liquid Chromatography Tandem Mass Spectrometry Analysis

**DOI:** 10.3390/molecules23010225

**Published:** 2018-01-20

**Authors:** Yan-Fang Yang, Lei Zhang, Xiu-Wei Yang

**Affiliations:** State Key Laboratory of Natural and Biomimetic Drugs, Department of Natural Medicines, School of Pharmaceutical Sciences, Peking University, No. 38, Xueyuan Road, Haidian District, Beijing 100191, China; yangyanfang@bjmu.edu.cn (Y.-F.Y.); zhangyutian0619@163.com (L.Z.)

**Keywords:** *Angelicae Pubescentis Radix*, coumarins, rat cerebrospinal fluid, rat brain, UPLC-MS/MS

## Abstract

*Angelicae Pubescentis Radix* (APR) is a widely-used traditional Chinese medicine. Pharmacological studies have begun to probe its biological activities on neurological disorders recently. To assess the brain penetration and distribution of APR, a validated ultra-performance liquid chromatography tandem mass spectrometry method was applied to the simultaneous determinations of the main coumarins from APR in the rat cerebrospinal fluid (CSF) and brain after oral administration of APR extract, including psoralen, xanthotoxin, bergapten, isoimperatorin, columbianetin, columbianetin acetate, columbianadin, oxypeucedanin hydrate, angelol B, osthole, meranzin hydrate and nodakenetin. Most of the tested coumarins entered the rat CSF and brain quickly, and double-peak phenomena in concentration-time curves were similar to those of their plasma pharmacokinetics. Columbianetin had the highest concentration in the CSF and brain, while psoralen and columbianetin acetate had the largest percent of CSF/plasma and brain/plasma, indicating that these three coumarins may be worthy of further research on the possible nervous effects. Correlations between the in vivo brain distributions and plasma pharmacokinetics of these coumarins were well verified. These results provided valuable information for the overall in vivo brain distribution characteristics of APR and also for its further studies on the active substances for the central nervous system.

## 1. Introduction

*Angelicae Pubescentis Radix* (APR), the dried roots of *Angelica pubescens* Maxim. *f. biserrata* Shan et Yuan, is well known as Duhuo in traditional Chinese medicines. It has been widely used as an analgesic and anti-rheumatic drug for centuries in China [1]. Coumarins have been demonstrated to be the most abundant and bioactive compounds of APR, and more than 60 coumarins have been isolated and identified [2]. Previously, the in vitro intestinal absorptions across the human intestinal epithelial Caco-2 cell monolayer of main coumarins from APR [3,4,5] have been carried out in our group. The in vivo pharmacokinetics of sixteen coumarins in rat plasma after oral administration of APR extract (APRE) [6] have also been studied. All the test compounds were defined as well- or moderately-absorbed compounds. The systematic studies on the absorption characteristics of the major coumarins of APR laid the foundation to further reveal the pharmacodynamic actions of APR.

Recently, pharmacological studies have begun to probe the biological activities of APR on neurological disorders. The water or ethanol extracts of APR have been demonstrated to have central nervous system (CNS) activities [7], such as inhibition of the apoptosis of brain cells [8] and inflammation of central nerves of Alzheimer’s disease (AD) model rats [9], as well as protection from H_2_O_2_-induced SH-SY5Y cell injury [10]. The CNS activities of single coumarins from APR have also been reported. For example, osthole protects cortical neurons and SH-SY5Y cells against β-amyloid peptide injury [11] and traumatic brain injury [12]; scopoletin ameliorates age-impaired memory disorders [13]; while isoimperatorin [14] and xanthotoxin [15] can inhibit acetylcholinesterase activity for the treatment of AD. With the development research of APR on neurological disorders, it is necessary to carry out studies on the brain penetration and distribution of the main compounds of APR, which have not been reported up to date.

**Figure 1:**
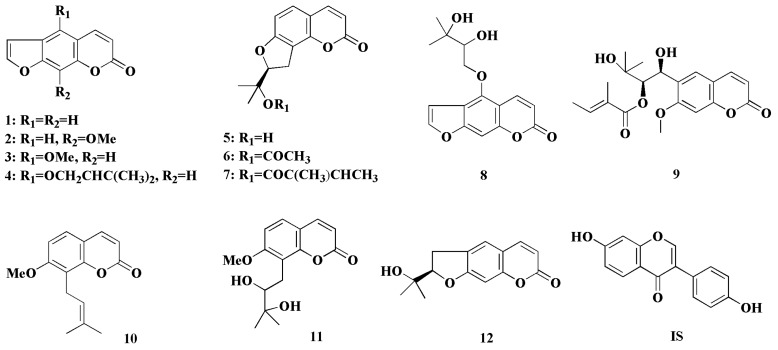
Chemical structures of main coumarins from *Angelicae Pubescentis Radix* (APR) and the internal standard. (**1**) psoralen, (**2**) xanthotoxin, (**3**) bergapten, (**4**) isoimperatorin, (**5**) columbianetin, (**6**) columbianetin acetate, (**7**) columbianadin, (**8**) oxypeucedanin hydrate, (**9**) angelol B, (**10**) osthole, (**11**) Meranzin hydrate, (**12**) nodakenetin, (internal standard (IS)) daidzein.

In the present work, the distributions of twelve coumarins from APR (chemical structures shown in Figure 1) in rat cerebrospinal fluid (CSF) and brain after oral administration of APRE were simultaneously determined by a reliable ultra-performance liquid chromatography tandem mass spectrometry (UPLC-MS/MS) method. The aim of the work was to systematically reveal the CSF and brain distributions of the main coumarins from APR and provide suitable explanations for the substances’ basis for its CNS bioactivities.

## 2. Results and Discussion

### 2.1. Analytical Method Validation

**Figure 2:**
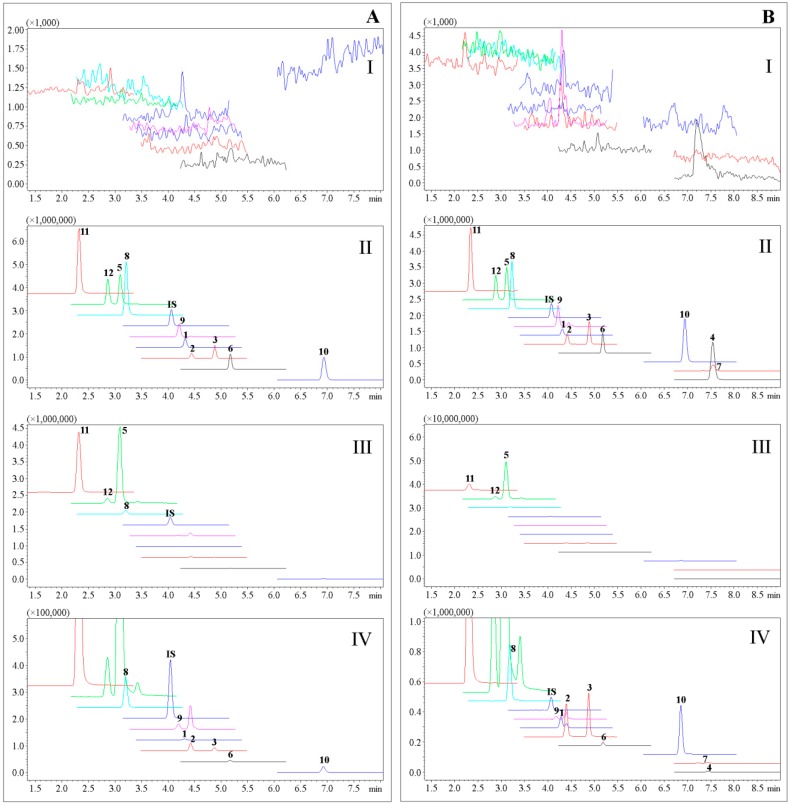
Multiple reaction monitoring (MRM) results of the analytes **1**–**12** and IS. (**A**) CSF samples: (I) blank CSF; (II) blank CSF spiked with analytes and IS of 20 ng/mL; (III) CSF sample at 2 h after oral administration of APR extract (APRE); (IV) enlarged view of III. (**B**) Brain samples: (I) blank brain; (II) blank brain spiked with analytes and IS of 20 ng/mL; (III) brain sample at 2 h after oral administration of APRE; (IV) enlarged view of III.

A reliable UPLC–MS/MS method was developed to simultaneously determine the distributions of **1**–**12** in rat CSF and brain. Daidzein was used as the internal standard (IS). The precursor ion, product ion, quadrupole 1 pre-rod bias (Q1), quadrupole 3 pre-rod bias (Q3), collision energy (CE) and dwell time (DT) were optimized and shown in Table 1. The chromatograms of blank CSF and brain homogenate samples showed no potential endogenous interferences (Figure 2).

Full method validations for the assay in blank CSF and rat brain were carried out according to the Guidance for Industry Bioanalytical Method Validation from the U.S. Food and Drug Administration [16]. The calibration curve was constructed by plotting the peak area ratio of analyte versus IS (*y*) and analyte concentration (*x*). The lower limit of quantification (LLOQ) was determined at the lowest concentration on the calibration curve with a signal-to-noise ratio of 10:1. The regression data and LLOQs of twelve analytes in CSF and rat brain are given in Table S1, and calibration curves showed good linearity with the correlation coefficient (*r*) >0.9950.

The intra-day accuracy and precision were evaluated by determining the quality control (QC) samples at three different concentrations for five replicates in the same day, and the inter-day accuracy and precision were determined over three consecutive days. As shown in Tables S2 and S3, the precisions in CSF and rat brain were within the ranges of 1.55–12.67% and 2.23–13.51%, while the accuracy percentages in CSF and rat brain were 89.71–113.75% and 91.50–110.73%, respectively. The stability was evaluated by measuring the QC samples after three freeze-thaw cycles or storage at room temperature for 24 h and expressed as the mean of percentage remaining by comparing the results with those of freshly-prepared QC samples. The values were 86.27–106.76% in CSF and 85.30–105.83% in rat brain, respectively, indicating that the samples were stable during the sample storage and processing procedures (Tables S2 and S3).

The recovery of **1**–**12** was determined by comparing the peak areas of equal analytes in QC samples with those in methanol (MeOH) solutions. The recoveries in both CSF and rat brain were within the range of 81.53–109.12%. The matrix effects were evaluated by comparing the peak area of analytes added into the pre-extracted CSF and blank brain with those in MeOH solutions. Matrix effects for all compounds were acceptable with the values of 84.96–111.84% in CSF and 86.76–111.16% in rat brain, respectively (Tables S2 and S3). In summary, the results confirmed that the developed method was sufficiently reliable for the simultaneous assessments of the twelve compounds in CSF and rat brain samples.

### 2.2. Rat CSF Distribution

Rat CSF distributions of analytes were carried out at 15 min as the first time point. An earlier time point was not achievable because of the limitation of collection methods for CSF and brain samples.

**Figure 3:**
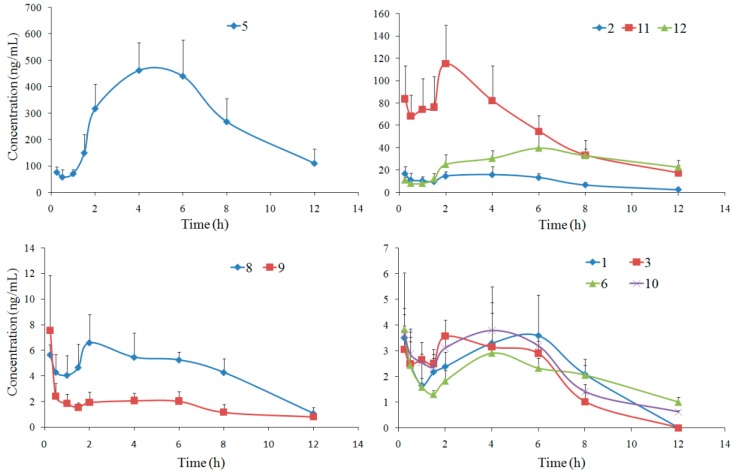
Mean concentration-time curves of ten compounds in rat CSF after oral administration of APRE (mean ± SD, *n* = 6). Coumarins **4** and **7** were unable to be determined since their MRM responses in CSF were under LLOQ.

CSF distributions of ten compounds were developed by the validated UPLC-MS/MS method, and their multiple reaction monitoring (MRM) results are shown in Figure 2. The concentration-time curves for the ten compounds are shown in Figure 3. Ten coumarins, except **4** and **7**, could be detected at 15 min after oral administration of APRE, indicating their rapid absorptions. The double-peak phenomena and concentration trends in rat CSF for the ten compounds were similar to those of reported plasma pharmacokinetics results [6]. Coumarin **5** had the highest content in CSF with a maximum concentration (C_max_) of (485.36 ± 91.40) µg/L and an area under concentration-time curve (AUC_0→∞_) of (4142.82 ± 602.33) µg/L·h, closely related to its high plasma C_max_ of (2240.32 ± 1031.92) µg/L [6]. Coumarins **4** and **7**, as well as bergaptol, imperatorin and umbelliferone, which were determined in the simultaneous pharmacokinetics studies, were also determined, but unable to be analyzed, since their MRM responses were under LLOQ. The low detections for these compounds may be related to their low concentration, small volumes and impossible enrichments of CSF.

The concentration-time data were fitted into the non-compartmental model based on statistical moment theory, and the pharmacokinetic parameters were calculated and listed in Table 2. The half-life (t_1/2_) values of most compounds, especially of coumarins **6** and **12**, were much longer than those in rat plasma [6], indicating that they worked longer in the CSF than in the plasma. The clearance (CL) values of these compounds varied widely, and Compound **5** with the smallest CL of 0.44 L/h/kg had the highest content in rat CSF.

### 2.3. Rat Brain Distribution

**Figure 4:**
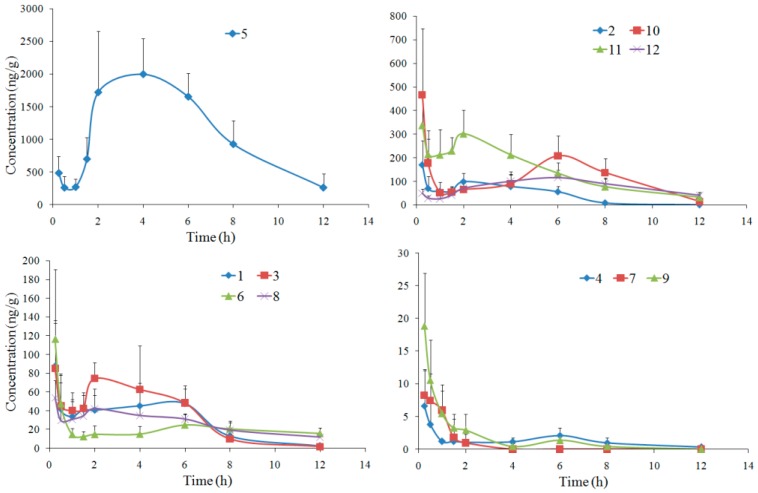
Mean concentration-time curves of twelve compounds in rat brain after oral administration of APRE (mean ± SD, *n* = 6).

Brain distributions of twelve compounds were also developed by the validated UPLC-MS/MS method. The concentration-time curves are shown in Figure 4, and the summaries of pharmacokinetic parameters are listed in Table 3. All twelve compounds could be detected at 15 min after oral administration of APRE, indicating that they entered the brain rapidly. Coumarin **7** was detected only within 4 h after oral administration, since its MRM response was under LLOQ at the following time points.

Compared with the previous simultaneous pharmacokinetics study in our group [6], double-peak phenomena of brain distribution for most tested compounds were similar to those of plasma pharmacokinetics, and the contents in brain were closely related to the plasma concentrations. The other three compounds, bergaptol, imperatorin and umbelliferone, were also determined for their brain distributions, but were unable to be analyzed, since their MRM responses were under LLOQ.

The t_1/2_ and CL values of most compounds were similar to those of in rat plasma [6]. As for coumarins **6** and **10**, t_1/2_ values in rat brain increased to (8.22 ± 4.54) and (5.36 ± 2.15) h, as well as the CL values decreased to (9.77 ± 5.25) and (13.96 ± 7.04) g/h/kg, respectively. As for coumarin **9**, however, the t_1/2_ value decreased from (6.63 ± 4.37) h in plasma to (2.77 ± 1.15) h in brain, and the CL value increased from (110.26 ± 39.27) g/h/kg in plasma to (301.26 ± 92.33) g/h/kg in brain. Thus, the eliminations of coumarins **6** and **10** slowed down in rat brain compared to in the plasma, while coumarin **9** was eliminated in the brain faster than in the plasma.

### 2.4. Relationship between the Pharmacokinetics and CSF/Brain Distribution

**Figure 5:**
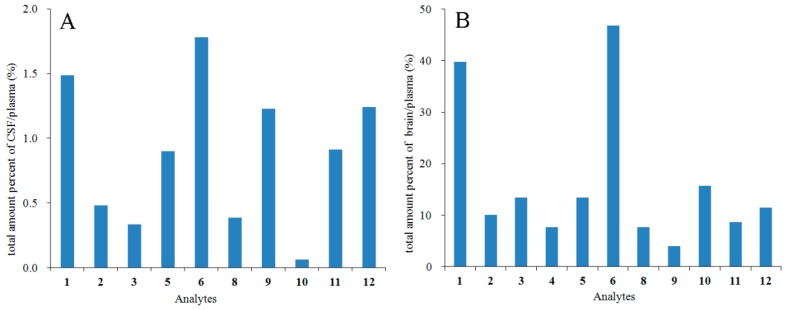
Total amount percentage of tested compounds in rat CSF (**A**) and brain (**B**) after oral administration of APRE.

A goal of CNS pharmaceutics is the estimation of the free drug in brain, including brain homogenate and CSF distribution [17], and the percent of AUC_brain_/AUC_plasma_ had been reported to represent the drug permeability across the blood-brain barrier (BBB) [18]. Since there was a slight difference in the units between the AUC_brain_ (µg/kg·h) and AUC_plasma_ (µg/L·h), the total amount percentage of CSF/plasma as (volume_CSF_ × AUC_CSF_)/(volume_blood_ × AUC_plasma_), as well as the total amount percentage of brain/plasma as (weight_brain_ × AUC_brain_)/(volume_blood_ × AUC_plasma_) were calculated to compare the CSF or brain distribution degrees with the pharmacokinetics [6] of these coumarins. The volumes of CSF and plasma in a 250-g rat were estimated to be about 480 µL [19] and 16 mL [20], as reported previously, and the average weight of rat brain in the study was 1.77 g. Thus the total amount percentage of CSF/plasma was calculated as (0.48 × AUC_0→∞/CSF_)/(16 × AUC_0→∞/plasma_), while the total amount percentage of brain/plasma was (1.77 × AUC_0→∞/brain_)/(16 × AUC_0→∞/plasma_). The results showed that the total amount percentage of CSF/plasma or brain/plasma were 0.06−1.78% and 4.02−46.81% (Figure 5). Coumarins **1**, **6**, **9** and **12** were the most distributed coumarins in the rat CSF with the total amount percentage values greater than 1%. Most compounds, except **4**, **8**, **9** and **11**, had total amount percentage values larger than 10% in the rat brain. Coumarins **1** and **6** were highly distributed both in the CSF and the brain, indicating that they might have had more possible CNS effects. As for Compounds **9**, **11** and **12**, they had relatively higher contents in the CSF than in the brain, while the distributions of **10** in the CSF and brain were the opposite. Perhaps the differences were related to the different pathways by which drugs in the plasma and CSF reached the brain [17].

A common measure of the degree of BBB penetration was the ratio of the steady-state concentrations of the drug molecule in the brain and in the blood, usually expressed as log BB [21]. The log P and polar surface area (PSA) values of the twelve analytes were calculated by Pallas 3.3.2.6 ADEM/Tox Software (Bal Harbor, Florida, FL, USA). The predicted log BB values (log BB_pred._) were calculated from the modified Clark’s Equation (1), and the experimental log BB values (log BB_expt._) were calculated from the tested data (2) [21]. The calculated equations of log BB_pred._ and log BB_expt._ are shown as follows, and the results are shown in Table 4:

log BB_pred._ = 0.152logP − 0.0148PSA + 0.139 (1)

log BB_expt._ = log (AUC_0→∞/brain_/AUC_0→∞/plasma_) (2)

It had been reported that within the range from −2.00–+1.00, compounds with log BB values >0.3 could well distribute to the brain, while those <−1.0 were poorly distributed [22]. The values of log BB_pred._ and log BB_expt._ generally agreed with the well or moderately BBB distributed characteristics of all analytes. Coumarins **8** and **9** had the smallest values of log BB_pred._ and log BB_expt._, indicating that their brain distributions were the weakest among these twelve coumarins. The log BB results of coumarins **8** and **9** were consistent with their smallest total amount percentage in brain/plasma in Figure 5. On the other hand, the largest log BB_expt._ values of coumarins **1** and **6** also coincided with their highest brain distributions.

### 2.5. Relationship between the In Vivo and In Vitro BBB Penetration

The in vitro BBB permeability of coumarins from APR had been studied previously in our group, and the bidirectional parameter apparent permeability coefficient (*P_app_*) values of Compounds **1**–**4**, **6**–**7** and **10** had been reported [23]. They were classified as well-absorbed compounds across the in vitro BBB model, except for coumarin **4** as a moderately-absorbed compound. 

In this study, the CSF and brain distribution results of the corresponding compounds (Figure 5) were consistent with the in vitro BBB permeabilities mentioned above, and the in vivo and in vitro results all confirmed the well-absorbed characteristics across BBB of these coumarins. The in vivo distribution result of coumarin **7** was absent in Figure 5 because of its low MRM responses under LLOQ both in CSF and brain, which was owed to its low plasma concentration [6]. Among the other six coumarins with in vitro *P_app_* values, coumarin **4** had the smallest total amount percentage of brain/plasma and was not detected in the rat CSF, in accordance with its moderate permeation across BBB in vitro.

## 3. Experimental Section

### 3.1. Chemicals and Reagents

APR was the same batch, and APRE was prepared as we had previously reported [6]. Psoralen (**1**), xanthotoxin (**2**), bergapten (**3**), isoimperatorin (**4**), columbianetin (**5**), columbianetin acetate (**6**), columbianadin (**7**), oxypeucedanin hydrate (**8**), angelol B (**9**), osthole (**10**) and nodakenetin (**12**) were isolated by the authors, as described in our previous reports [24,25,26]. Meranzin hydrate (**11**) and daidzein (IS, structure given in Figure 1) were obtained from Shanghai Yuanye Bio-Tech. Co. Ltd. (Shanghai, China) and the National Institutes for Food and Drug Control (Beijing, China), respectively. The purities of all standards were above 98%. MeOH and acetonitrile (ACN) were of LC-MS grade (J. T. Backer, Center Valley, PA, USA). Formic acid was of HPLC grade (Dikma, Lake Forest, CA, USA). De-ionized water was prepared with a Milli-Q water purification system (Millipore, Bedford, MA, USA). Artificial cerebrospinal fluid was purchased from Beijing Leagene Biotech. Co. Ltd. (Beijing, China). Other chemicals and solvents used were of analytical grade.

### 3.2. Preparation of Calibration Standards and Quality Control Solutions

Compounds **1**–**12** and IS were respectively dissolved in MeOH to prepare the stock solutions. Appropriate aliquots of individual stock solutions were mixed together to prepare mixed stock solutions. The solution was serially diluted with blank CSF or blank brain homogenate to prepare calibration standard solutions with IS (20 ng/mL finally). QC solutions were individually prepared at high, medium and low concentration levels, according to the linear ranges of coumarins **1**–**12**. All the samples were stored at −20 °C.

### 3.3. Preparation of Rat CSF and Brain Samples

Each weighed brain sample was homogenized with a 2-fold volume of normal saline solution. IS solution (10 μL, 200 ng/mL) was added to 300 μL of the homogenate. The mixture was then extracted with a 3-fold volume of ethyl acetate. The mixture was vortexed for 1 min and centrifuged at 16,000× *g* for 10 min. The supernatant was transferred out and dried under a gentle constant flow of nitrogen. The residue was then dissolved with 100 μL of MeOH and ultrasonically (power 250 W, frequency 40 KHz) extracted for 10 min. After centrifugation at 16,000× *g* for 10 min, the supernatant was drawn out into a clean vial, and 5 μL of each were injected into the UPLC–MS/MS system for analysis.

Aliquots of 50 μL CSF samples with IS (10 μL, 200 ng/mL) were extracted with 600 μL ethyl acetate. Subsequent steps were identical to those described above for the treatment of rat brain samples.

### 3.4. UPLC-MS/MS Analysis

The UPLC-MS/MS system consisted of a Shimadzu Nexera UPLC instrument connected to a Shimadzu LCMS-8050 mass spectrometer via the electrospray ionization interface (Shimadzu Corp., Kyoto, Japan). Samples were separated on an ACQUITY UPLC BEH RP18 column (2.1 × 100 mm, 1.7 µm, Waters, Milford, MA, USA) equipped with a BEH RP_18_ guard column (2.1 × 5 mm, 1.7 µm, Waters, Milford, MA, USA). The column temperature was 30 °C, and the flow rate was 0.4 mL/min. The sample injection volume was 5 µL. The mobile phases consisted of 0.1% (*v*/*v*) formic acid in water (A) and ACN (B). The LC gradient elution program and mass working parameters were the same as our previous report [6]. Compounds **1**–**12** were quantified in positive ion mode with MRM mode. The precursor ion, product ion, Q1, Q3, CE and DT were optimized.

### 3.5. Animals and Dosing

The animal study was in accordance with the guidelines for the Care and Use of Laboratory Animals in Beijing and approved by the Animal Care and Use Committee of Peking University (Approval No. LA 2014161, approved on 27 February 2014). Male Sprague-Dawley rats (body weight 250 ± 10 g) were supplied by the Laboratory Animal Center of Peking University Health Science Center (license No. SCXK (Jing) 2016-0010, Beijing, China). Animals were housed under controlled environmental conditions (temperature 24 ± 2 °C; relative humidity 60 ± 5%; 12 h light/dark cycle).

The time schedule included 9 time-points, and six rats were sampled at each time-point. The rats were fasted overnight with free access to water before the experiment. APRE was suspended with the normal saline and orally administered to the rats at a single dose of 4 g/kg. The animals had free access to water during the experiment.

### 3.6. Rat CSF and Brain Collections

Rats were anesthetized by an intraperitoneal dose of chloral hydrate (350 mg/kg) at 0.25, 0.5, 1, 1.5, 2, 4, 6, 8, 12 h after oral administration of APRE. A dorsal midline cutaneous incision was made, and the muscle at the craniocervical junction was bluntly dissected to expose the atlanto-occipital membrane. The cisterna magna was punctured with a thin syringe needle, and a microinjector connected with the needle was used to collect the interior CSF carefully [27]. Clear CSF was transferred to a clean microtube and frozen at −80 °C until further use. Blood contaminated samples were excluded.

Cardiac perfusion was carried out quickly with physiological saline after CSF collection at each time-point. The rat brains were separated, weighed and then stored at −80 °C until treatment.

### 3.7. Data Analysis

Results were expressed as the mean ± standard deviation (SD). The UPLC-MS/MS data were processed with Labsolutions 5.65 software (Shimadzu Corp., Kyoto, Japan). The pharmacokinetic parameters were calculated with the Drug and Statistics 2.0 software (Mathematical Pharmacology Professional Committee of China, Shanghai, China).

## 4. Conclusions

A validated UPLC-MS/MS method was applied for the distribution assessments of twelve coumarins in the rat CSF and brain after oral administration of APRE. Most coumarins entered the rat CSF and brain quickly. Columbianetin had the highest concentration in both rat CSF and brain, while psoralen and columbianetin acetate had the largest percent of CSF/plasma and brain/plasma. The possible CNS effects of these three coumarins may be worthy of further research. Correlations between the in vivo brain distributions and the in vivo pharmacokinetics or in vitro BBB permeabilities of these coumarins were well verified. The results provided valuable information for the overall in vivo brain distribution characteristics of the coumarins from APR and also for its further studies of CNS active substances.

## Figures and Tables

**Table 1:** The MW, precursor ion, product ion, quadrupole 1 pre-rod bias (Q1), quadrupole 3 pre-rod bias (Q3), collision energy (CE), dwell time (DT) and retention time (RT) of twelve coumarins and IS.

Compound	MW	Precursor Ion	Product Ion	Q1 (V)	Q3 (V)	CE (V)	DT (msec)	RT (min)
**1**	186	187	131.1	−14	−24	−25	47	4.3
**2**	216	217	202.1	−16	−21	−21	47	4.4
**3**	216	217	89.1	−26	−16	−49	47	4.9
**4**	270	271	203.0	−20	−21	−15	51	7.5
**5**	246	247	175.1	−19	−18	−23	47	3.1
**6**	288	289	229.1	−22	−24	−10	51	5.2
**7**	328	329	229.1	−30	−24	−12	51	7.5
**8**	304	305	203.0	−24	−21	−22	47	3.2
**9**	376	377	191.0	−28	−19	−39	47	4.2
**10**	244	245	189.1	−18	−19	−15	51	6.9
**11**	278	261	189.0	−20	−19	−18	47	2.3
**12**	246	247	175.1	−19	−18	−24	47	2.8
IS	254	255	199.1	−19	−20	−24	100	4.1

**Table 2:** Pharmacokinetic parameters of ten analytes * in rat CSF after oral administration of APRE (mean ± SD, *n* = 6).

Compound	AUC_0→t_ µg/L·h	AUC_0→∞_ µg/L·h	t_1/2z_ h	T_max_ h	CL_z/F_ L/h/kg	V_z/F_ L/kg	C_max_ µg/L
**1**	20.40 ± 6.16	51.28 ± 17.49	3.52 ± 2.04	4.67 ± 1.63	22.80 ± 9.30	75.49 ± 28.52	4.49 ± 1.05
**2**	120.75 ± 19.00	129.46 ± 15.65	2.57 ± 0.89	1.50 ± 1.48	19.88 ± 2.56	76.01 ± 35.34	20.28 ± 5.13
**3**	27.08 ± 2.26	41.34 ± 5.77	6.72 ± 1.95	1.46 ± 0.84	34.09 ± 5.69	319.33 ± 65.49	3.95 ± 0.24
**5**	3374.64 ± 604.02	4142.82 ± 602.33	4.06 ± 2.42	5.00 ± 1.10	0.44 ± 0.06	2.52 ± 1.42	485.36 ± 91.4
**6**	24.16 ± 3.44	43.63 ± 22.07	7.68 ± 3.28	0.30 ± 0.11	102.68 ± 34.34	1030.65 ± 388.37	4.49 ± 1.91
**8**	54.03 ± 8.20	73.64 ± 11.84	6.32 ± 1.95	2.00 ± 1.14	11.02 ± 2.02	97.70 ± 24.17	7.47 ± 1.69
**9**	20.01 ± 3.35	28.17 ± 3.94	7.02 ± 2.51	0.38 ± 0.31	251.74 ± 39.96	2507.26 ± 843.89	7.65 ± 4.14
**10**	27.70 ± 2.88	30.51 ± 1.53	3.19 ± 1.16	2.83 ± 2.84	766.97 ± 36.55	3573.87 ± 1393.27	4.31 ± 1.49
**11**	675.91 ± 107.84	753.71 ± 141.64	3.37 ± 0.75	2.17 ± 0.98	2.24 ± 0.38	10.70 ± 1.87	128.51 ± 20.19
**12**	330.91 ± 43.19	624.72 ± 249.36	6.69 ± 2.25	6.33 ± 0.82	1.89 ± 0.61	16.76 ± 2.68	43.50 ± 5.98

* The MRM responses of coumarins **4** and **7** in CSF were under LLOQ, so their pharmacokinetic parameters were unable to be calculated.

**Table 3:** Pharmacokinetic parameters of eleven analytes * in rat brain after oral administration of APRE (mean ± SD, *n* = 6).

Compound	AUC_0→t_ µg/kg·h	AUC_0→∞_ µg/kg·h	t_1/2z_ h	T_max_ h	CL_z/F_ g/h/kg	V_z/F_ g/kg	C_max_ µg/g
**1**	351.63 ± 65.06	371.95 ± 69.76	2.15 ± 0.67	0.33 ± 0.13	2.88 ± 0.58	8.86 ± 3.08	99.93 ± 30.91
**2**	519.05 ± 151.70	732.88 ± 450.63	1.88 ± 0.96	0.33 ± 0.13	4.26 ± 1.71	16.48 ± 11.23	192.74 ± 74.07
**3**	418.20 ± 93.60	449.85 ± 97.52	2.34 ± 1.04	1.21 ± 1.53	3.22 ± 0.85	10.76 ± 4.73	104.59 ± 36.22
**4**	15.51 ± 4.82	21.56 ± 14.56	3.54 ± 1.35	0.33 ± 0.13	52.86 ± 24.79	245.44 ± 108.54	8.32 ± 4.75
**5**	13,438.17 ± 2016.06	16,664.04 ± 3662.67	3.92 ± 3.33	4.00 ± 1.79	0.11 ± 0.02	0.57 ± 0.39	2245.65 ± 638.71
**6**	251.98 ± 58.91	489.71 ± 222.11	8.22 ± 4.54	0.33 ± 0.13	9.77 ± 5.25	95.22 ± 25.54	128.03 ± 63.26
**8**	313.39 ± 59.25	397.36 ± 66.83	4.91 ± 0.91	0.96 ± 0.86	2.04 ± 0.34	14.45 ± 3.95	62.56 ± 13.78
**9**	21.06 ± 8.70	25.06 ± 8.03	2.77 ± 1.15	0.29 ± 0.10	301.26 ± 92.33	1299.16 ± 842.00	19.74 ± 7.52
**10**	1327.91 ± 341.99	2105.63 ± 1100.15	5.36 ± 2.15	0.35 ± 0.14	13.96 ± 7.04	60.18 ± 25.11	448.58 ± 206.25
**11**	1752.16 ± 249.82	1948.10 ± 305.84	3.64 ± 1.40	1.29 ± 1.50	0.86 ± 0.13	4.46 ± 1.60	396.56 ± 63.09
**12**	933.83 ± 66.51	1560.07 ± 730.91	6.27 ± 3.19	5.33 ± 1.03	0.779 ± 0.26	6.30 ± 2.22	129.97 ± 15.50

* The data points of coumarin **7** in rat brain are not enough to calculate the parameters because of its zero content from 4 h, so its pharmacokinetic parameters were unable to be calculated.

**Table 4:** The log P, polar surface area (PSA) and log BB (brain, blood) values of the twelve analytes.

Analytes	1	2	3	4	5	6	7 *	8	9	10	11	12
log P	2.08	2.17	2.17	3.70	1.67	2.54	4.28	1.11	2.04	4.08	1.10	1.59
PSA	39.44	48.67	48.67	48.67	55.76	61.83	61.83	89.13	102.29	35.53	75.99	55.76
log BB_pred._	−0.13	−0.25	−0.25	−0.02	−0.43	−0.39	−0.13	−1.01	−1.06	0.23	−0.82	−0.44
log BB_expt._	0.56	−0.04	0.08	−0.16	0.08	0.63	–	−0.16	−0.44	0.15	−0.10	0.01

* The pharmacokinetic parameters of coumarin **7** were unable to be calculated, so its log BB_expt._ was absent.

## Supplementary Materials

UPLC-MS/MS method validation results for the assay are available online as Supplementary Materials.
