# Supplementary material for: Distribution Assessments of Coumarins from Angelicae Pubescentis Radix in Rat Cerebrospinal Fluid and Brain by Liquid Chromatography Tandem Mass Spectrometry Analysis
Source: Molecules. 2018 Jan 20;23(1):225. doi: 10.3390/molecules23010225 (PMC6017000; doi:10.3390/molecules23010225)
Supplement: Supplementary file 1 [file molecules-23-00225-s001.pdf]

Supplementary data for:

Distribution Assessments of Coumarins from *Angelicae Pubescentis Radix* in Rat Cerebrospinal Fluid and Brain by Liquid Chromatography Tandem Mass Spectrometry Analysis

Yan-Fang Yang, Lei Zhang, Xiu-Wei Yang*

State Key Laboratory of Natural and Biomimetic Drugs, Department of Natural Medicines, School of Pharmaceutical Sciences, Peking University, No. 38, Xueyuan Road, Haidian District, Beijing 100191, China; E-Mails: yangyanfang@bjmu.edu.cn (Y.-F.Y.); zhangyutian0619@163.com (L. Z); xwyang@bjmu.edu.cn (X.-W.Y.)

* Correspondence: xwyang@bjmu.edu.cn; Tel.: +86 10 82801569; Fax: +86 10 82802724.

Table S1. Calibration curves and LLOQ of twelve coumarins in rat brain and aCSF

Biosamples	Analytes	Calibration curves	Correlation coefficient (*r*)	Linear range (ng/ml)	LLOQ (ng/ml)
Brain	**1**	$y = 0.0220x + 0.0763$	0.9994	2.0–200	2.0
	**2**	$y = 0.0391x + 0.2428$	0.9996	1.0–500	1.0
	**3**	$y = 0.0216x + 0.0341$	0.9994	1.0–200	1.0
	**4**	$y = 0.1544x + 0.0207$	0.9957	0.3–200	0.3
	**5**	$y = 0.0736x + 7.6627$	0.9959	0.1–3000	0.1
	**6**	$y = 0.0264x + 0.0532$	0.9992	0.3–200	0.3
	**7**	$y = 0.0108x + 0.0711$	0.9950	1.5–200	1.5
	**8**	$y = 0.1137x + 0.1525$	0.9996	0.3–200	0.3
	**9**	$y = 0.0348x + 0.0395$	0.9995	0.3–200	0.3
	**10**	$y = 0.1295x + 0.5175$	0.9975	0.3–200	0.3
	**11**	$y = 0.1331x + 1.5667$	0.9988	0.1–500	0.1
	**12**	$y = 0.0744x + 0.3038$	0.9997	0.2–500	0.2
aCSF	**1**	$y = 0.0128x + 0.0030$	0.9973	2.0–100	2.0
	**2**	$y = 0.0165x - 0.0297$	0.9954	1.0–300	1.0
	**3**	$y = 0.0131x - 0.0079$	0.9954	1.0–100	1.0
	**5**	$y = 0.0632x - 0.0465$	0.9967	0.1–800	0.2
	**6**	$y = 0.0264x - 0.0101$	0.9994	0.3–100	0.3
	**8**	$y = 0.1019x + 0.0132$	0.9982	0.3–100	0.3
	**9**	$y = 0.0376x + 0.0007$	0.9982	0.3–100	0.3
	**10**	$y = 0.1338x - 0.0526$	0.9971	0.3–100	0.3
	**11**	$y = 0.1119x + 0.3983$	0.9959	0.1–300	0.1
	**12**	$y = 0.0492x - 0.0114$	0.9966	0.2–300	0.2

Table S2. Precision, accuracy, matrix effect, recovery and stability of ten analytes in aCSF (n = 5)

Analytes	Concentration (ng/mL)	Precision RSD (%)		Accuracy (%)		Matrix effect(%)	Recovery (%)	Stability (%)	
		Intra-day	Inter-day	Intra-day	Inter-day			Freeze-thaw	room temperature
**1**	10	9.49	12.69	96.59	103.76	93.42±8.91	90.98±10.72	96.14±6.21	97.19±5.40
	40	5.91	8.04	102.91	103.27	95.49±10.85	92.10±3.27	96.98±5.79	98.36±3.57
	80	9.46	12.03	94.58	96.93	88.32±6.16	93.90±9.73	86.27±13.45	94.12±6.94
**2**	5	4.72	12.87	104.19	108.35	103.16±11.12	88.75±7.51	97.35±8.04	96.39±6.08
	120	6.30	12.44	96.84	92.68	89.15±3.29	87.72±5.95	96.65±5.56	98.95±5.53
	240	3.49	8.15	98.13	102.27	89.32±13.78	87.76±7.65	92.14±5.06	98.24±5.62
**3**	5	3.25	10.34	98.25	101.37	86.08±5.49	87.66±2.59	92.34±7.48	106.76±8.57
	40	3.70	4.54	101.55	102.89	91.59±7.69	109.12±7.46	93.24±6.30	94.61±4.23
	80	2.55	7.26	101.93	104.35	89.51±6.89	84.86±4.89	97.23±5.17	99.54±7.55
**5**	5	7.95	13.51	98.01	99.44	97.16±12.32	92.45±3.63	96.35±9.69	97.23±8.25
	320	2.86	7.51	97.92	98.84	96.17±8.22	89.44±6.43	93.46±8.56	93.93±2.94
	640	4.34	6.44	104.40	107.77	104.90±2.07	90.38±2.89	93.87±4.82	99.45±9.62
**6**	5	5.52	6.39	101.55	103.30	106.49±7.19	86.71±3.78	96.34±7.65	98.70±7.26
	40	4.33	7.28	97.58	98.82	97.56±5.33	85.25±4.32	98.19±6.63	95.51±7.41
	80	2.89	8.29	101.37	106.33	88.09±8.92	86.91±4.26	98.77±3.53	95.92±7.85
**8**	5	4.09	7.61	98.92	101.78	96.44±8.87	87.09±2.24	97.57±5.59	96.96±3.22
	40	2.47	6.26	93.35	95.53	89.75±9.45	89.34±11.00	94.73±7.34	95.09±3.26
	80	3.17	6.83	97.50	98.19	89.10±8.12	85.64±7.77	95.96±5.37	103.37±5.33
**9**	5	6.13	9.55	106.91	110.73	96.92±13.16	97.65±2.56	95.14±6.53	97.45±4.87
	40	5.14	8.51	91.50	94.81	101.59±9.87	90.48±4.73	95.36±7.29	95.67±3.47
	80	4.93	9.51	95.61	97.90	84.95±6.38	87.17±7.38	96.89±4.68	96.66±9.22
**10**	5	4.55	9.01	104.58	109.68	104.15±6.07	106.43±6.15	94.43±7.49	95.92±3.20
	40	4.71	6.81	97.04	95.83	109.82±6.84	92.80±13.03	95.90±8.12	94.93±5.05
	80	2.23	8.63	95.72	101.52	87.97±9.72	101.50±6.99	97.71±2.91	93.50±6.38
**11**	5	4.38	9.66	104.12	108.57	111.84±9.52	88.35±9.52	96.16±10.07	92.02±3.40
	120	3.51	7.10	96.29	97.40	87.91±10.64	97.81±9.39	95.58±7.24	99.09±5.56
	240	3.23	7.16	95.70	97.85	96.45±8.39	87.84±9.66	94.40±4.03	103.10±9.99
**12**	5	3.05	6.81	101.43	104.49	99.43±8.84	86.12±3.63	101.36±5.89	102.33±8.37
	120	4.06	9.47	97.27	98.62	91.73±12.98	87.44±5.05	92.78±5.37	101.14±5.08
	240	3.07	7.81	99.74	103.77	105.36±6.80	84.29±7.10	95.63±4.23	99.91±7.69

Table S3. Precision, accuracy, matrix effect, recovery and stability of twelve analytes in rat brain (*n* = 5)

Analytes	Concentration (ng/mL)	Precision RSD (%)		Accuracy (%)		Matrix effect(%)	Recovery (%)	Stability (%)	
		Intra-day	Inter-day	Intra-day	Inter-day			Freeze-thaw	room temperature
**1**	10	4.41	5.90	98.09	105.90	86.76±5.07	81.53±9.58	97.11±8.58	101.36±7.46
	80	4.94	6.08	96.75	97.60	105.89±6.27	87.25±6.87	96.22±7.77	99.53±12.87
	160	3.70	4.17	98.63	99.15	95.81±13.22	89.06±5.30	96.30±8.22	96.99±3.28
**2**	5	6.43	12.67	97.41	108.96	96.64±5.52	104.81±3.44	86.06±4.97	87.23±8.49
	200	3.27	7.89	104.55	105.94	106.35±8.95	84.54±5.30	92.93±10.30	91.44±4.50
	400	5.40	9.94	97.50	98.88	101.79±10.76	84.08±5.41	85.90±6.94	88.73±6.7
**3**	5	7.08	12.29	99.82	105.90	87.72±7.32	88.80±2.94	89.97±6.29	88.02±2.73
	80	1.55	3.81	98.75	99.24	106.61±8.34	86.33±4.86	85.30±8.42	86.01±3.16
	160	5.12	6.37	96.26	97.94	102.54±12.92	90.62±9.13	90.70±9.46	86.84±6.63
**4**	5	7.98	9.02	98.77	99.55	93.71±7.38	85.10±3.84	104.96±13.66	91.59±10.74
	80	6.86	7.40	99.80	104.68	98.23±10.16	88.75±9.92	93.10±4.03	88.65±10.02
	160	3.93	10.12	99.09	101.80	111.16±9.69	93.28±12.83	89.68±3.96	86.85±3.37
**5**	5	9.66	10.90	109.57	113.75	91.80±10.90	107.47±2.78	105.83±7.01	97.88±10.06
	1200	7.22	9.67	103.26	111.03	87.48±7.85	88.79±11.27	91.69±3.42	89.46±3.10
	2400	9.22	11.73	92.99	94.87	102.56±5.72	94.47±6.04	87.85±9.09	91.21±6.60
**6**	5	5.40	11.35	97.80	102.52	91.52±9.90	86.91±5.59	91.80±9.93	95.28±9.39
	80	4.99	6.86	104.90	101.45	94.61±3.73	86.75±3.35	93.45±7.15	96.74±2.26
	160	3.37	5.94	102.47	103.56	96.88±9.82	92.41±9.75	96.02±5.61	98.33±3.12
**7**	10	2.55	9.21	91.39	89.71	98.39±5.80	84.77±4.91	101.64±6.63	96.82±4.84
	80	5.02	8.44	96.03	94.74	92.78±8.70	90.89±12.46	94.79±6.28	92.73±3.53
	160	5.92	9.41	98.02	97.85	90.15±5.34	85.62±2.71	90.93±4.84	91.16±4.37
**8**	5	4.89	9.49	108.03	107.06	105.16±9.67	88.44±7.12	96.57±11.87	97.72±9.35
	80	5.00	11.19	104.82	97.68	99.31±10.46	89.51±5.73	94.94±10.27	96.02±6.30
	160	5.19	7.15	97.75	96.89	109.02±11.44	94.34±1.89	102.42±7.10	97.99±6.44
**9**	5	8.28	11.15	106.51	107.08	97.84±11.62	85.96±3.20	101.51±11.81	96.24±7.79
	80	4.72	9.76	103.06	104.11	96.00±9.10	87.14±5.10	95.20±9.40	97.77±8.23
	160	5.06	11.17	97.09	99.87	89.82±9.95	85.50±10.07	102.98±8.06	96.20±7.07
**10**	5	6.46	9.03	99.17	95.26	93.29±7.20	94.78±8.82	95.53±4.36	99.12±3.06
	80	6.22	8.23	97.90	102.58	93.96±7.52	86.80±6.41	98.42±10.02	101.64±8.78
	160	6.45	9.34	99.75	103.65	95.96±11.08	96.82±12.37	98.13±6.85	96.43±4.95
**11**	5	6.72	11.31	94.95	105.48	105.12±6.43	89.81±2.09	94.71±4.63	97.21±8.86
	200	2.73	7.66	97.79	99.04	108.01±11.12	94.70±7.32	97.28±10.13	96.58±7.80
	400	5.15	11.90	99.66	102.91	106.44±12.95	98.35±5.48	104.50±7.46	95.82±9.16
**12**	5	2.93	5.15	98.93	103.29	93.59±9.07	84.86±2.33	98.18±2.46	94.83±5.41
	200	4.72	8.24	99.49	105.48	102.05±10.48	90.07±7.83	101.08±9.25	97.69±5.38
	400	3.03	7.11	97.41	99.60	89.18±11.29	93.11±4.28	96.81±6.42	95.56±2.64
